# Rapid synthesis of manganese dioxide nanoparticles for enhanced biocompatibility and theranostic applications

**DOI:** 10.1039/d4ra06995a

**Published:** 2025-01-30

**Authors:** Xueting Wang, Xiaoqing Yang, Xiaoqing Yi, Xuehong Min, Yongmei Jia

**Affiliations:** a Department of Respiratory and Critical Care Medicine, The Affiliated Jiangning Hospital of Nanjing Medical University Nanjing 211100 China; b College of Pharmacy, Gannan Medical University Ganzhou 341000 China keyi0115@126.com; c Equine Science Research and Horese Doping Control Laboratory, Hubei Provincial Engineering Research Center of Racing Horse Detection and Application Transformation, Wuhan Business University Wuhan 430056 China mxh201270267@163.com; d College of Chemistry and Chemical Engineering, Lingnan Normal University Zhanjiang 524048 China jiayongmei214@126.com

## Abstract

Manganese dioxide (MnO_2_), lauded for its biocompatibility and distinctive optical and physical characteristics, has become an indispensable material in the biomedical field, showing immense potential in disease detection, treatment, and prevention. Particularly, the ability of MnO_2_ nanoparticles to oxidize glutathione (GSH) to its oxidized form has positioned them as pivotal players in GSH sensing. However, conventional preparation methods, whether top-down or bottom-up, often result in nanoparticles that require multi-step processing and modification to achieve good dispersion in physiological conditions, which is both time-consuming and complex. To address this, a rapid and efficient method was developed for producing well-dispersed and stable MnO_2_ nanoparticles using tannic acid to reduce potassium permanganate. The polyphenolic structure of tannic acid not only facilitates the reduction process but also enhances the dispersibility of the nanoparticles in biological environments. In addition, PEG could improve the stability of MnO_2_ nanoparticles and also reduce their size. Moreover, we demonstrate the application of these nanoparticles in a colorimetric assay for GSH detection, leveraging their ability to react with GSH to produce Mn^2+^. Furthermore, these nanoparticles were utilized in a colorimetric assay for GSH detection, harnessing their reactivity with GSH to generate Mn^2+^. Beyond this, the MnO_2_ nanoparticles exhibit potential for the loading of a spectrum of molecules, including small molecules, peptides, DNA, RNA, and proteins, through electrostatic interactions, π–π stacking, and the inherent reactivity of polyphenols. This groundbreaking strategy heralds a new era for MnO_2_ in the realm of theranostic agent delivery, offering a promising avenue for enhancing diagnostic accuracy and therapeutic efficacy in biomedical applications.

## Abstract 2

Rapid preparation of MnO_2_ and by using MnO_2_ for colorimetric detection of GSH.

## Graphical abstract

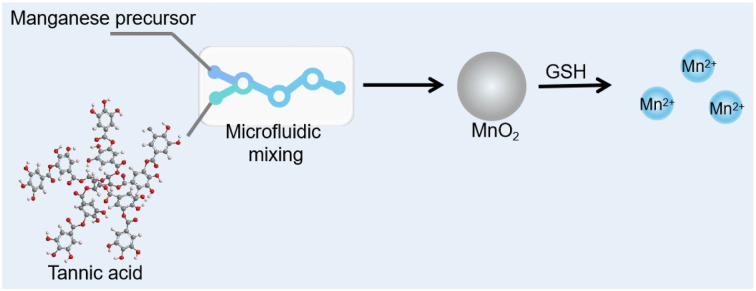

## 1. Introduction

MnO_2_ is an important transition metal oxide that has garnered significant attention due to its diverse crystalline forms, excellent chemical stability, and superior chemical performance.^1–3^ MnO_2_ nanoparticles, with its smaller particle size and larger surface area, exhibits more optimized material properties and a broader range of applications compared to traditional MnO_2_.^4–7^ The characteristics and applications of MnO_2_ are widespread, particularly in the medical and diagnostic fields where MnO_2_ nanoparticles play a crucial role.^8,9^ In terms of synthesis methods, nanoscale MnO_2_ can be prepared through various routes, including the hydrothermal method,^10^ sol–gel process,^11^ chemical precipitation,^12^ and solid phase synthesis.^13^ The hydrothermal method allows for the synthesis of nanoscale MnO_2_ under high temperature and pressure, yielding materials with high purity, good dispersion, and complete crystalline forms.^14^ The chemical precipitation method is simple to operate and cost-effective, making it suitable for mass production, but it may result in poor uniformity of the products. Solid-state synthesis, on the other hand, is a simple, efficient, and low-cost approach.^15^

Despite significant progress in the synthesis and biomedical application of nanoscale MnO_2_, ensuring the long-term stability of these nanoparticles under physiological conditions is crucial for preserving their diagnostic and therapeutic efficacy.^16–18^ To accomplish this, various strategies have been devised by researchers, notably the use of highly biocompatible polymers, such as polyethylene glycol (PEG), for surface modification of these nanoparticles.^19^ Preparation methods often involve using Mn^2+^ or KMnO_4_ as raw materials, and the fabrication of MnO_2_ nanoparticles for medical and diagnostic applications typically requires multiple steps and a longer duration. Therefore, there is an urgent need to develop a simple and rapid method for preparing well-dispersed and physiologically stable MnO_2_ nanoparticles.

Tannic acid (TA), a polyphenolic compound derived from plants and approved by the FDA as a food additive, is celebrated for its remarkable attributes. These include excellent biocompatibility, biodegradability, responsiveness to stimuli, and self-healing capabilities.^20^ TA is renowned for its exceptional antioxidant capabilities and free radical scavenging activity, effectively safeguarding cells, tissues, and organs from oxidative stress-induced damage. TA serving as reducing and capping/stabilizing agents has been used to synthesis noble metal nanoparticles including gold nanoparticles,^21^ and silver nanoparticles.^22^ The multiple hydroxyl functional groups of TA reduce metallic ions to atomic forms, and subsequently aggregate into nuclei, sparking crystal growth culminating in the formation of metal nanostructures. Utilizing TA as template, MnO_2_ nanomaterials with flower-like and spherical morphologies through a straightforward and eco-friendly approach.^23^ Nonetheless, larger nanoparticles in solution are generally less stable and more susceptible to aggregation and sedimentation. Consequently, mitigating nanoparticle aggregation and bolstering their stability in aqueous media are essential for advancing their practical applications.

Colorimetric and fluorescent biosensors have indeed garnered significant interest in the detection of pathogens due to their inherent advantages, such as simplicity, speed, and cost-efficiency. These sensors do not require expensive machinery or specialized training, making them suitable for routine examinations across various applications. In the context of glutathione (GSH) detection, Wang *et al.* have utilized MnO_2_-phenol formaldehyde resin for detecting GSH in blood serum.^24^ However, they noted that larger nanoparticles in the probe exhibit poor stability due to aggregation in water, which can impact the reliability of the detection. To address this, a label-free fluorescent sensing strategy based on G-quadruplex formation for the selective detection of glutathione has been developed, offering a high degree of sensitivity and selectivity without the need for additional labels, making it a cost-effective and convenient approach for GSH detection.^25^ However, it is important to consider the use of Hg^2+^ in such strategies, as mercury is a well-known toxic heavy metal with potential environmental hazards. The development and application of such biosensors highlights the ongoing innovation in the field, with a focus on enhancing detection capabilities while minimizing environmental and health risks.

**Scheme 1:**
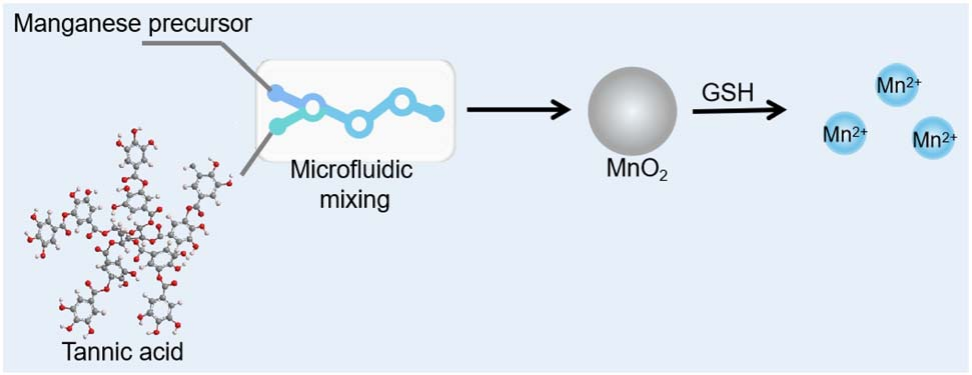
Schematic illustration of rapid preparation of MnO_2_ and using MnO_2_ for colorimetric detection of GSH.

In this study, we innovatively employ tannic acid as reducing agent to rapidly synthesize MnO_2_ nanoparticles at room temperature through a one-step process, while the good aqueous solubility property of PEG was utilized to enhance the stability of colloidal nanoparticles (Scheme 1). This simple preparation method not only boosts production efficiency but also ensures that the nanoparticles maintain their stability in physiological environments, laying a foundation for their application in the medical field. The color of the solution shifted from a vibrant purple to a rich brown during the synthesis. In the presence of glutathione (GSH), MnO_2_ could be reduced to Mn^2+^, marked by a color change from brown to colorless. This transformation has allowed us to develop a colorimetric nanosensor for the detection of the biomarker GSH using these MnO_2_ nanoparticles. The sensor has been successfully applied to the detection of GSH levels in serum samples, demonstrating high sensitivity and selectivity. Additionally, the carboxyl and phenolic hydroxyl groups on tannic acid provide a rich platform for the functionalization of MnO_2_ nanoparticles.^26^ Various agents such as electrostatic interactions, π–π stacking, and covalent modifications can be loaded to MnO_2_ nanoparticles, transforming these nanoparticles into multifunctional diagnostic and therapeutic platforms. This research not only showcases the innovative application of tannic acid in nanomaterial synthesis but also paves the way for the development of novel biomedical diagnostic tools and therapeutic carriers. Future studies will further explore the potential of these nanoparticles in disease diagnosis and treatment, as well as their safety and efficacy *in vitro* and *in vivo*.

## 2. Experimental

### 2.1 Reagents

Tannic acid and KMnO_4_ were obtained from Guangfu Reagent Company (Tianjin, China). Polyethylene glycol (PEG) was sourced from Sigma-Aldrich (St. Louis, MO, USA). All solvents and reagents used were of analytical grade.

### 2.2 Preparation of MnO_2_ nanoparticles

Tannic acid was dispersed in a phosphate-buffered saline (PBS) solution at pH 7.4 to create a homogeneous solution. This step is crucial for ensuring that the tannic acid is evenly distributed, which is essential for the subsequent redox reaction. An aqueous KMnO_4_ solution was carefully mixed with the tannic acid dispersion in the PBS solution to initiate the redox reaction. The careful control of the mixing ratio and the reaction conditions is vital for the controlled synthesis of MnO_2_ nanoparticles. The reaction was conducted in a microfluidic chip (INano™ Optimux), which allows for precise control over the reaction conditions and the mixing of reagents. A 10 mg ml^−1^ PEG2000 solution and a 20 mg ml^−1^ tannic acid dispersion were combined within the INano™ Optimux microfluidic chip. The addition of PEG2000 serves to stabilize the nanoparticles and prevent aggregation. The mixture was then combined with a 2 mg per ml KMnO_4_ aqueous solution at varying ratios to optimize the synthesis process. The resulting MnO_2_ nanoparticles were collected through PBS washing to remove any unreacted precursors and by-products, followed by centrifugation at 8000 g for three cycles to ensure the complete separation and purification of the nanoparticles.

### 2.3 Transmission electron microscopy (TEM)

The MnO_2_ nanoparticles solution nanoparticles were applied to a carbon-coated copper grid for 10 minutes to allow adsorption. The grid was then stained with uranyl acetate and examined under a Thermo Talos 120 C microscope in Waltham, USA.

### 2.4 Dynamic light scattering and zeta potential measurements

The size distribution, polydispersity index, and zeta potential of the MnO_2_ nanoparticles were determined using a ZetaSizer Nano series Nano-ZS from Malvern Instruments Ltd in Malvern, UK. The measurements were performed at 25 °C, with sample dilution in deionized water as required.

### 2.5 Detection of GSH using MnO_2_ NPs

The absorbance of the solution was measured using a Mettler Toledo UV5 UV-vis spectrophotometer. Glutathione (GSH) at various concentrations was added to the MnO_2_ solution and incubated for 10 min at room temperature. The solutions were then diluted and mixed with ultrapure water, transferred to a quartz cuvette, and their absorbance was measured. The kinetic response was assessed by monitoring the absorbance changes of MnO_2_ nanoparticles in the presence of GSH over different incubation periods.

### 2.6 Detection of GSH in human serum samples

All experiments involving human serum were conducted in accordance with the guidelines and standards established by the National Health Commission of the People's Republic of China. Informed consents were obtained from human participants of this study. The human serum samples were collected and analyzed with the approval of the Medical Ethics Committee of the Affiliated Jiangning Hospital of Nanjing Medical University (2023-03-119-K01). After centrifugation at 10 000 rpm for 10 minutes, the supernatant was diluted 30-fold and mixed with GSH at concentrations of 10, 20, and 30 μM in a centrifuge tube containing MnO_2_ nanoparticles. The mixture was incubated for 10 minutes at room temperature, and the absorbance was subsequently measured using UV-vis spectroscopy.

## 3. Results and discussion

### 3.1 Design and preparation of MnO_2_ nanoparticles

**Fig. 1:**
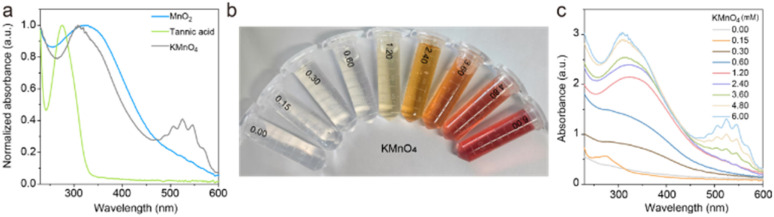
(a) Normalized UV-vis spectra of MnO_2_, tannic acid and KMnO_4_. (b) Digital photograph and (c) UV-vis spectra of the mixture with different concentration of KMnO_4_ ranging from 0 to 6.0 mM.

Tannic acid, known for its antioxidant properties due to its propensity to undergo oxidation, can readily be oxidized by potassium permanganate (KMnO_4_) to form manganese dioxide nanoparticles (MnO_2_ NPs). Its chelating ability with metal ions enhances dispersibility. Polyethylene glycol 2000 (PEG2000), used as a stabilizing agent, helps reduce the particle size of MnO_2_ NPs. The rapid synthesis of MnO_2_ NPs is illustrated in Scheme 1. In a standard preparation procedure, a manganese precursor, tannic acid, and PEG2000 are combined in a microfluidic system, followed by centrifugation to remove larger particles, yielding MnO_2_ NPs. As depicted in Fig. 1a, the absorption spectrum of tannic acid shows peaks at approximately 275 nm, while KMnO_4_ exhibits bands at 307 nm, 507 nm, 524 nm, and 545 nm. The prepared MnO_2_ NPs display a new band at 335 nm, confirming the successful synthesis of the nanoparticles. During the refinement of the MnO_2_ NPs synthesis, the concentration of KMnO_4_ was carefully optimized. Fig. 1b demonstrates that the introduction of KMnO_4_ induces a significant color change in the solution, shifting from transparent to a deeper hue. The color intensity of solution increases with KMnO_4_ concentration, peaking at 2.4 mM. Beyond this concentration, the solution turns a distinct purple brown, suggesting a partial conversion of KMnO_4_ to MnO_2_. Additionally, the absorbance spectra indicates that the signal at 335 nm rises with increasing KMnO_4_ concentration. When the KMnO_4_ concentration exceeds 2.4 mM, the signal at 335 nm continues to grow. The presence of bands at 507 nm, 524 nm, and 545 nm, characteristic absorption peaks of KMnO_4_, further suggests that the reaction is not complete. The optimal ratio for preparing MnO_2_ NPs from PEG2000, KMnO_4_, and tannic acid is established at 100 : 8 : 2.5. This mild synthesis route, facilitated on a microfluidic chip, promises scalability and translational potential from laboratory to clinical settings.

**Fig. 2:**
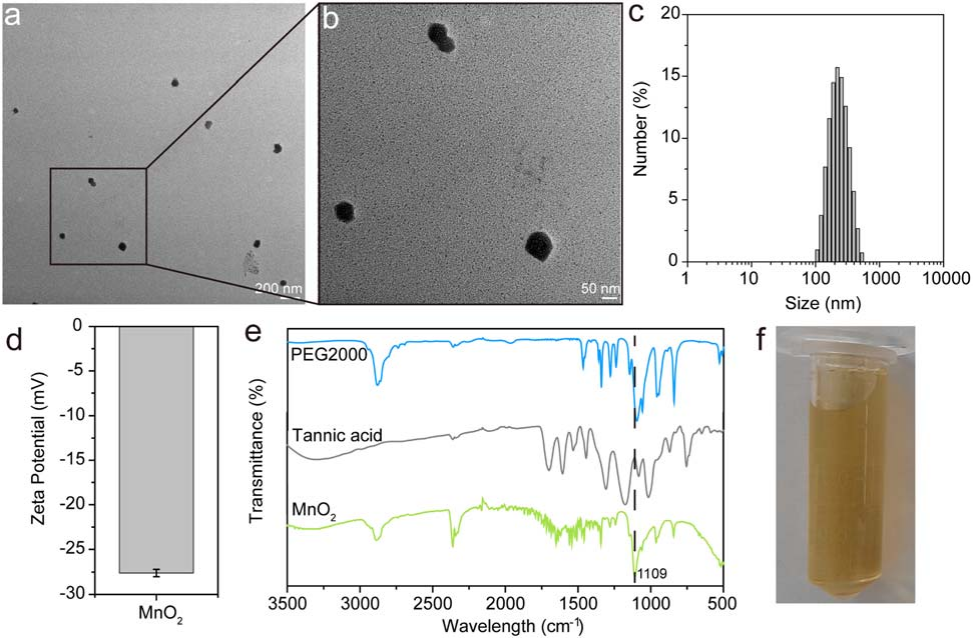
(a and b) Representative TEM image of MnO_2_ nanoparticles. (c) Particle size distribution and (d) zeta potential for MnO_2_ nanoparticles by DLS. (e) FT-IR spectra of PEG2000, tannic acid and MnO_2_ nanoparticles. (f) Digital photograph of MnO_2_ nanoparticles.

The morphological characteristics of MnO_2_ NPs were examined using transmission electron microscopy (TEM) and dynamic light scattering (DLS). Fig. 2a and b illustrate that the TEM analysis confirms the spherical shape and uniform dispersion of the MnO_2_ NPs, with particle diameters ranging from 20 to 80 nm. The DLS measurements determine the average hydrodynamic diameter of the MnO_2_ NPs to be 206 nm, accompanied by a zeta potential of approximately −27.6 ± 0.4 mV. This surface charge is attributed to the presence of tannic acid and PEG, which not only stabilize the nanoparticles but also endow them with excellent stability in physiological conditions, rendering them suitable for biomedical applications. Furthermore, the FT-IR spectra in Fig. 2e reveal the presence of characteristic peaks of PEG and tannic acid within the MnO_2_ NPs, identified at 3311, 2359, 2098, 1278, 1097, 962, and 840 cm^−1^. Notably, the band at 1109 cm^−1^, corresponding to the stretching vibration of the C–O–C bonds in PEG, experiences a redshift, which is attributed to the interaction between the MnO_2_ NPs and the vibrational modes of PEG. In the lower frequency region, a broad band from 800 to 500 cm^−1^ is observed, indicative of the Mn–O and Mn–O–Mn vibrations within the [MnO_6_] octahedral structure.^1^ The visual inspection of the prepared nanoparticles, as depicted in Fig. 2f, shows a brown color, which is characteristic of MnO_2_. Collectively, these findings confirm the successful synthesis of MnO_2_ NPs with the desired morphological and chemical properties.

### 3.2 Detection of GSH using MnO_2_

**Fig. 3:**
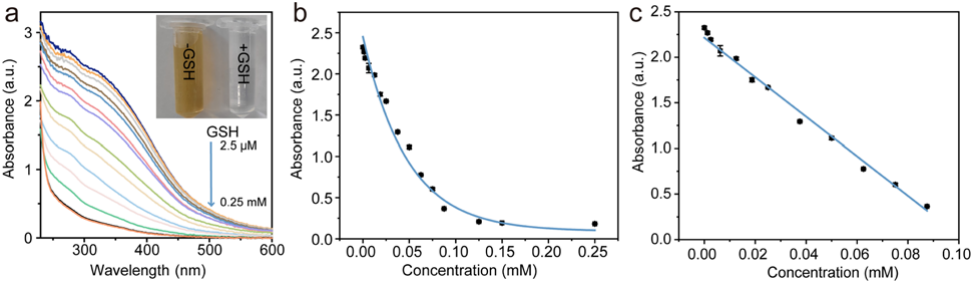
Analysis of the MnO_2_ nanoparticles response to GSH. (a) UV-vis absorption spectra of MnO_2_ nanoparticles in the presence of different concentrations of GSH. (b) Relationship between absorbance and the target concentration ranging from 0 to 0.25 mM. (c) The calibration curve establishes a linear relationship between absorbance and GSH concentration for the range of 2.5 to 87.5 μM.

Manganese dioxide nanoparticles (MnO_2_ NPs), with their unique optical and electrochemical characteristics, have become promising candidates for the detection of glutathione (GSH).^27–30^ Utilizing a colorimetric approach, this method provides a visual assessment of GSH levels through observable color changes. MnO_2_ NPs were synthesized that exhibit a distinctive yellow color and an absorption spectrum spanning from 300 nm to 500 nm. These nanoparticles serve as a sensing platform, leveraging their absorbance and color alterations for GSH detection. The redox interaction between MnO_2_ and GSH leads to the conversion of the yellow MnO_2_ into colorless Mn^2+^, allowing for the effective monitoring of GSH concentrations. Under the optimal conditions established, a simple and potent visual assay was developed for the sensitive and precise detection of GSH, with a signal reduction of 92.1%. As depicted in Fig. 3a, a progressive reduction in ultraviolet (UV) absorption is observed upon the introduction of glutathione (GSH), indicating MnO_2_ is degraded into coloress Mn^2+^. The absorption plateaus when the GSH concentration reaches 0.25 mM. Fig. 3b illustrates that the absorbance signal decreases almost linearly with increasing GSH concentrations up to 87.5 μM. The calibration curve (Fig. 3c) for GSH concentrations is defined by the equation:*A* = 2.21 − 21.7[GSH] (*R*^2^ = 0.991, [GSH] = 2.5 − 87.5 μM)

This calibration equation provides a quantitative framework for determining GSH levels in aqueous solutions. The detection limit (DL) was calculated using the formula: DL = 3.3*δ*/*k*, where *δ* is the standard deviation of the blank sample measurements and *k* is the slope of the calibration curve. The calculated DL for the MnO_2_ nanoprobe was 1.4 μM, indicating that the MnO_2_ nanoprobe shows potential as an outstanding optical sensor for the quantitative analysis of GSH in biological samples.

### 3.3 Selectivity of MnO_2_ NPs for GSH detection

**Fig. 4:**
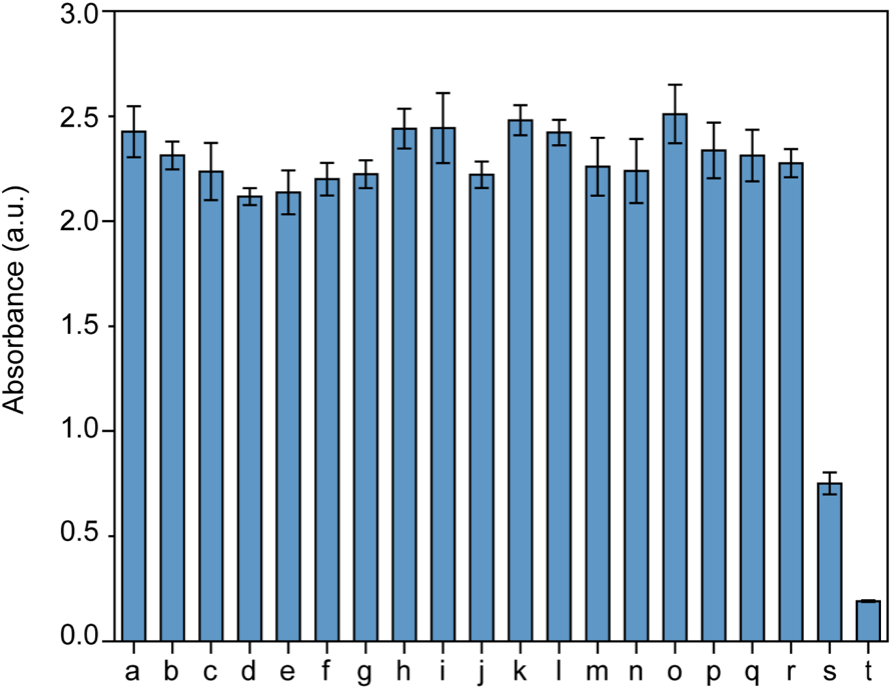
Assessing the selectivity of MnO_2_ nanoparticles for GSH over other potential interferences. (a) KCl, (b) NaCl, (c) MgSO_4_, (d) CoCl_2_, (e) MnCl_2_, (f) CaCl_2_, (g) Na_2_SO_4_, (h) KI, (i) KF, (j) K_2_SO_3_, (k) K_2_SO_4_, (l) K_2_S, (m) glutamine, (n) alanine, (o) lysine, (p) guanine, (q) threonine, (r) glucose, (s) ascorbic acid, (t) GSH.

The selectivity and specificity of MnO_2_ NPs for the colorimetric detection of GSH were rigorously evaluated to ensure the method's reliability. To assess the selectivity, we performed tests with a variety of common interferences to assess the specificity of MnO_2_ NPs. Notably, the presence of GSH led to a significant reduction in the absorbance signal of MnO_2_ NPs, as evidenced in Fig. 4. Conversely, the addition of other biological ions and amino acids did not result in a discernible decrease in the absorbance signal, demonstrating the high selectivity of MnO_2_ NPs toward GSH. The selectivity of our detection system is largely due to the distinctive redox properties of MnO_2_, which selectively engages with GSH owing to its distinctive thiol group. While glucose, a reducing sugar, fails to interact with MnO_2_ under identical conditions. Notably, only reducing biomolecules such as ascorbic acid (AA) and GSH elicit significant absorption signals, whereas amino acids and electrolytes do not cause a marked increase in the absorption signal. Although AA can trigger a response in this system, its concentrations in biological systems are typically lower than those of GSH, which are in the millimolar range. This specificity allows for the accurate detection of GSH without interference from other biological molecules. Consequently, MnO_2_ NPs have been established as a selective nanoprobe for the detection of GSH in human serum, offering a reliable and interference-free analytical approach.

### 3.4 Practical application

In order to substantiate the practical application and reliability of our GSH detection method, human serum samples were utilized for real-world testing. We employed the standard addition method to incorporate a range of GSH standard solution concentrations into the serum samples. Following this, the absorbance was measured, and the recovery rates were determined, with a summary of the results presented in Table 1. The GSH content in diluted human serum is 22.3 and 16.4 μM, the GSH concentration in diluted FBS is 4.5 μM, while in goat serum it is 13.8 μM. To ensure the accuracy of our detection method, we introduced an internal standard and assessed the recovery rates. The recovery rates, which indicate the method's accuracy in recovering the added GSH, ranged from 98.7% to 109.6%. This high recovery rate confirms the effectiveness of our method in detecting GSH in complex biological matrices and demonstrates its potential for use in various biological and clinical applications. These outcomes demonstrate the high practicality of our sensing system for GSH detection, offering a robust and efficient technique suitable for various real-world scenarios. The successful application of MnO_2_ NPs to serum samples highlights its potential for wider utilization to accurately measure GSH in different biological matrices, such as fetal bovine serum and mouse serum, further showcases the method's versatility and adaptability, making it an invaluable tool for a broad array of experimental settings.

**Table 1:** Determination of GSH in diluted serum by employing MnO_2_ NPs

Sample	Found in sample (μM)	Added (μM)	Totally found (μM)	Recovery rate (%)
Human serum	22.3 ± 2.9	10	34.8 ± 2.2	107.7
		20	45.2 ± 3.9	106.9
		30	56.8 ± 3.4	108.6
	16.4 ± 3.2	10	27.5 ± 1.2	104.2
		20	38.5 ± 1.1	105.7
		30	52.2 ± 6.0	112.5
FBS	4.5 ± 1.6	10	15.1 ± 2.4	103.9
		20	25.1 ± 3.2	102.3
		30	37.2 ± 4.1	107.8
	3.4 ± 0.9	10	13.7 ± 2.3	102.1
		20	24.7 ± 5.6	105.9
		30	36.5 ± 4.5	109.6
Mouse serum	13.8	10	25.8 ± 3.3	108.5
		20	35.9 ± 3.7	106.2
		30	43.5 ± 3.5	99.4
	15.4	10	25.9 ± 1.7	102.1
		20	34.9 ± 5.2	98.7
		30	46.9 ± 4.5	103.4

## 4. Conclusions

The utilization of tannic acid for the synthesis of MnO_2_ nanoparticles marks a noteworthy advancement in nanomedicine. These nanoparticles have been demonstrated to be a potent signalling platform for the colorimetric detection of GSH, a molecule pivotal to cellular mechanisms including redox homeostasis, detoxification, and immune response. The method provides a platform, offering simplicity, sensitivity, and selectivity. Their facile synthesis, cost-effectiveness, and excellent stability and reproducibility position them as strong candidates for clinical diagnostics and point-of-care testing. This innovative approach underscores the transformative impact of nanotechnology in enhancing the precision of diagnostics and the effectiveness of therapeutics within the biomedical field.

## Data availability

The data are included within the article.

## Author contributions

Xueting Wang coordinated the study, performed the experiments, and drafted the manuscript. Xiaoqing Yang and Xiaoqing Yi contributed to the experimental design and data analysis. Yongmei Jia and Xuehong Min performed the statistical analysis, contributed to the editing of the manuscript and supervised the project and secured funding. All authors read and approved the final manuscript.

## Conflicts of interest

There are no conflicts to declare.

## References

[cit1] Jia Y., Yi X., Li Z., Zhang L., Yu B., Zhang J., Wang X., Jia X. (2020). Talanta.

[cit2] Zhang J. T., Zhao Z. H., Xia Z. H., Dai L. M. (2015). Nat. Nanotechnol..

[cit3] Gao Z. F., Ogbe A. Y., Sann E. E., Wang X., Xia F. (2018). Talanta.

[cit4] Li Y. J., Liu Y. F., Liu D. L., Zhao X. H., Yin Y. Y., Zhu S. L., Shen X. Y., Chen H. L., Ji S. L., Hao Y. W. (2024). ACS Appl. Nano Mater..

[cit5] Limaye A. S., Rananaware P., Ghosh A., Rajashekarreddy T., Raghavendrarao N., Brahmkhatri V., Hegde R. V., Dateer R. B. (2024). Acs Appl. Bio Mater..

[cit6] Wang J. H., Zhou Y. P., Lv Y., Feng J. F., Wang Z. P., Cai G. F. (2024). Small.

[cit7] Wang X. G., Shen M., Sun Y. Y., Tang Q. Y., Du L., Yang S., Zou H. B., Zhao X., Chen X. J., Li H. S., Li J. R., Wang X. H., Lao L. F., Yang D. Y., Gu B., Liu P. F. (2024). Nano Today.

[cit8] Lin L. S., Song J. B., Song L., Ke K. M., Liu Y. J., Zhou Z. J., Shen Z. Y., Li J., Yang Z., Tang W., Niu G., Yang H. H., Chen X. Y. (2018). Angew. Chem. Int. Ed..

[cit9] Yang G. B., Xu L. G., Chao Y., Xu J., Sun X. Q., Wu Y. F., Peng R., Liu Z. (2017). Nat. Commun..

[cit10] Qu Z. P., Fan R., Wang Z., Wang H., Miao L. (2015). Appl. Surf. Sci..

[cit11] Wang X. Y., Wang X. Y., Huang W. G., Sebastian P. J., Gamboa S. (2005). J. Power Sources.

[cit12] Pan T. T., Deng H., Kang S. Y., Zhang Y., Lian W., Zhang C. B., He H. (2021). Chem. Eng. J..

[cit13] Zeng X. C., Zhang X. J., Liu S., Yang H., Tao Z. L., Liang J. (2019). Sci. China: Chem..

[cit14] Wang F., Zheng Y., Chen Q., Yan Z., Lan D., Lester E., Wu T. (2024). Coordin. Chem. Rev..

[cit15] Nam H. S., Kwon J. S., Kim K. M., Ko J. M., Kim J. D. (2010). Electrochim. Acta.

[cit16] Choi D., Kang T. G., Kim T., Moon C. W., Choi M., Kim D., Kim T., Oh Y., Jung S., Lee Y., Lee S., Hong J., Ha S. J. (2024). Nano Today.

[cit17] Haidar L. L., Bilek M., Akhavan B. (2024). Small.

[cit18] Kaur N., Gautam P., Nanda D., Meena A. S., Shanavas A., Prasad R. (2024). Bioconjugate Chem..

[cit19] Luo Y. J., Zheng F., Gao Y. Y., Chen W. Y., Xue X. R., Xiao C. J., Wei K. (2024). Acs Appl. Nano Mater..

[cit20] Suzawa M., Miranda D. A., Ramos K. A., Ang K. K. H., Faivre E. J., Wilson C. G., Caboni L., Arkin M. R., Kim Y. S., Fletterick R. J., Diaz A., Schneekloth J. S., Ingraham H. A. (2015). Elife.

[cit21] Wang C. M., Hou P. X., Zhao Y. M., Shi C., Zhang J. G., Wu A. P., Liu C., Cheng H. M. (2023). Nano Res..

[cit22] Jayeoye T. J., Supachettapun C., Muangsin N. (2023). Sci. Rep..

[cit23] Sorouri F., Gholibegloo E., Mortezazadeh T., Kiani S., Foroumadi A., Firoozpour L., Khoobi M. (2023). Sci. Rep..

[cit24] Wang X. D., Wang D., Guo Y. L., Yang C. D., Liu X. Y., Iqbal A., Liu W. S., Qin W. W., Yan D., Guo H. C. (2016). Biosens. Bioelectron..

[cit25] Zhao J. J., Chen C. F., Zhang L. L., Jiang J. H., Shen G. L., Yu R. Q. (2013). Analyst.

[cit26] Bigham A., Rahimkhoei V., Abasian P., Delfi M., Naderi J., Ghomi M., Moghaddam F. D., Waqar T., Ertas Y. N., Sharifi S., Rabiee N., Ersoy S., Maleki A., Zare E. N., Sharifi E., Jabbari E., Makvandi P., Akbari A. (2022). Chem. Eng. J..

[cit27] He D. G., Yang X. X., He X. X., Wang K. M., Yang X., He X., Zou Z. (2015). Chem. Commun..

[cit28] Ma H., Li X. R., Liu X. Y., Deng M., Wang X. D., Iqbal A., Liu W. S., Qin W. W. (2018). Sens. Actuators, B.

[cit29] Ma H., Liu X. Y., Wang X. D., Li X. R., Yang C. D., Iqbal A., Liu W. S., Li J. P., Qin W. W. (2017). Microchim. Acta.

[cit30] Wang C. Y., Gao Y., Hu S., Zhu A. N., Ying Y., Guo X. Y., Wu Y. P., Wen Y., Yang H. F. (2022). Biosens. Bioelectron..

